# Data from the European registry for patients with McArdle disease (EUROMAC): functional status and social participation

**DOI:** 10.1186/s13023-023-02825-z

**Published:** 2023-07-25

**Authors:** Walaa Karazi, Renata S. Scalco, Mads G. Stemmerik, Nicoline Løkken, Alejandro Lucia, Alfredo Santalla, Andrea Martinuzzi, Marinela Vavla, Gianluigi Reni, Antonio Toscano, Olimpia Musumeci, Carlyn V. Kouwenberg, Pascal Laforêt, Beatriz San Millán, Irene Vieitez, Gabriele Siciliano, Enrico Kühnle, Rebecca Trost, Sabrina Sacconi, Hacer Durmus, Biruta Kierdaszuk, Andrew Wakelin, Antoni L. Andreu, Tomàs Pinós, Ramon Marti, Ros Quinlivan, John Vissing, Nicol C. Voermans, EUROMAC Consortium

**Affiliations:** 1 Neuromuscular Center Nijmegen, Department of Neurology, 910, Donders Institute for Brain, Cognition and Behaviour, Radboud University Nijmegen Medical Center, P.O. Box 9101, 6500 HB Nijmegen, The Netherlands; 2 MRC Centre for Neuromuscular Diseases, UCL Institute of Neurology, National Hospital, London, UK; 3 Copenhagen Neuromuscular Center, Section 8077, , Rigshospitalet, University of Copenhagen, 2100 Copenhagen, Denmark; 4 Faculty of Sport Sciences, Universidad Europea de Madrid, Madrid, Spain; 5 Instituto de Investigación Hospital, 12 de Octubre (imas12), Madrid, Spain; 6 Universidad Pablo de Olavide, Seville, Spain; 7 Departments of Neurorehabilitation, IRCCS Medea Scientifc Insitute, Conegliano-Pieve Di Soligo, Italy; 8 Department of Information Technology, Autonomous Province of Bolzano, Bolzano, Italy; 9 Neurology and Neuromuscular Diseases Unit, Department of Clinical and Experimental Medicine, University of Messina, Messina, Italy; 10 Neurology Department, Raymond Poincaré University Hospital, APHP, Garches, France; 11 Pathology Department, Alvaro Cunqueiro Hospital, Vigo, Spain; 12 Rare Diseases and Pediatric Medicine Research Group, Galicia Sur Health Research Institute (IIS Galicia Sur), SERGASUVIGO, Vigo, Spain; 13 Neurology and Neuromuscular Diseases Unit, Department of Clinical and Experimental Medicine, University of Pisa, Pisa, Italy; 14 Department of Neurology, Heimer Institute for Muscle Research, University Hospital Bochum, Bochum, Germany; 15 Peripheral Nervous System and Muscle Department, CHU Nice, Université Côte D’Azur, Institute for Research On Cancer and Aging of Nice (IRCAN), INSERM U1081, CNRS UMR 7284, Faculty of Medicine, Université Côte D’Azur (UCA), Nice, France; 16 Istanbul Faculty of Medicine, Istanbul University, Istanbul, Turkey; 17 Department of Neurology, Medical University of Warsaw, Warsaw, Poland; 18 Association for Glycogen Storage Disease (UK), Bristol, UK; 19 EATRIS, European Infrastructure for Translational Medicine, 1081 HZ Amsterdam, The Netherlands; 20 Biomedical Network Research Centre on Rare Diseases (CIBERER), Instituto de Salud Carlos III, and Research Group on Neuromuscular and Mitochondrial Diseases, Vall d’Hebron Research Institute, Universitat Autònoma de Barcelona, Barcelona, Catalonia Spain

**Keywords:** McArdle disease, Glycogen storage disease V, Rare diseases, International registry, Health care

## Abstract

**Background:**

The European registry for individuals with GSD5 and other muscle glycogenosis (EUROMAC) was launched to register rare muscle glycogenosis in Europe, to facilitate recruitment for research trials and to learn about the phenotypes and disseminate knowledge about the diseases. A network of twenty collaborating partners from eight European countries and the US contributed data on rare muscle glycogenosis in the EUROMAC registry.

**Methods:**

Following the initial report on demographics, neuromuscular features and comorbidity (2020), we here present the data on social participation, previous and current treatments (medication, supplements, diet and rehabilitation) and limitations. Furthermore, the following questionnaires were used: Fatigue severity scale (FSS), WHO Disability Assessment Scale (DAS 2.0), health related quality of life (SF36) and International Physical Activity Questionnaire (IPAQ).

**Results:**

Of 282 participants with confirmed diagnoses of muscle glycogenosis, 269 had GSD5. Of them 196 (73%) completed all questionnaires; for the others, the data were incomplete. The majority, 180 (67%) were currently working. Previous medical treatments included pain medication (23%) and rehabilitation treatment (60%). The carbohydrate-rich diet was reported to be beneficial for 68%, the low sucrose diet for 76% and the ketogenic diet for 88%. Almost all participants (93%) reported difficulties climbing stairs. The median FSS score was 5.22, indicating severe fatigue. The data from the WHODAS and IPAQ was not of sufficient quality to be interpreted.

**Conclusions:**

The EUROMAC registry have provided insight into the functional and social status of participants with GSD5: most participants are socially active despite limitations in physical and daily life activities. Regular physical activity and different dietary approaches may alleviate fatigue and pain.

## Introduction

Glycogen storage disease type 5, GSD5 (McArdle disease) is a rare metabolic myopathy with a prevalence of approximately 1:100,000 [1]. It is caused by inherited deficiency of muscle glycogen phosphorylase, also known as myophosphorylase. GSD5 is characterized by physical activity intolerance manifesting as muscle fatigue, cramping and pain, during vigorous activities [1]. If continuing activity at the same intensity, individuals are at risk for rhabdomyolysis and myoglobinuria [2]. GSD5 is rare and has an autosomal recessive inheritance pattern and a variable presentation. As a consequence, many participants experienced a long diagnostic delay [4]. This is expected to improve with the wide availability of diagnostic next-generation sequencing [31]. Once diagnosed, symptomatic treatment is often not provided.

The European registry for individuals with GSD5 and other muscle glycogenosis (EUROMAC) was launched to raise awareness of diagnostic accuracy of muscle GSDs, improve care, and collect important clinical and epidemiological data for use in future clinical trials by Scalco et al. [3, 4] The first two EUROMAC reports focused on the design of the registry and the clinical and genetic characteristics and comorbidities. The main findings were a high frequency of fixed weakness, normal CK values in a minority of participants, body mass index above background population and a high prevalence of hypothyroidism and coronary heart disease. [3, 4] We here present the additional data collected in the EUROMAC registry, including scores on social participation, previous and current treatments, functional limitations, fatigue, health disability, quality of life and physical activity.

## Methods

### EUROMAC registry

The registry was designed under the guidance and consensus of EUROMAC members at specific meetings during the first months of the EUROMAC project. EUROMAC members were twenty collaborating partners from eight European countries (Denmark, France, Germany, Greece, Italy, Spain, Turkey and United Kingdom) and USA. The Netherlands and Poland joined the project later and contributed individuals to the registry. The registry obtained the approval of all local Institutional review Boards for patient entry via the registry website (www.registryeuromac.eu).

The technical setup and data security for the registry are detailed in [3]. After review by people affected by GSDs and by a patient representative to ensure clarity [5], the participant information sheet and consent form were translated into the languages of the participating countries and adapted to follow local regulations.

### Participants and data entry

All participants consented in writing before inclusion in the registry. Any clinician working at a European institution was able to register on the EUROMAC platform and enter patient data after joining the EUROMAC partnership. Inclusion criteria were individuals with a diagnosis of one of the 14 known muscle GSDs either verified by genetic testing or enzymatic testing of the muscle biopsy. Individuals with Pompe disease were excluded as there was a well-established registry for this disease already. Following informed consent from the participant, data were pseudonymized and uploaded onto a safe, encrypted web-based registry. Recruited participants were able to log in, review their own information and complete selected sections with their personal experiences. None of the recruited participants or participating clinicians were allowed to see data from other participants.

Data entry items are shown in Table 3 by Pinos et al. [3] We here present the results collected in Sects. 6, 7 and 8 Data was only entered once, except if missing data was uploaded later. Data was based on clinical status and medical history at the time of data entry.

### Customized questions

Participants were asked about Limitations (Sect. 6), Previous/ongoing treatments (Drugs, Special diet, Supplements, Rehabilitation program, Other treatments; Sect. 7), and Services provided (including current work involvement and adaptations; Sect. 8). Drugs were categorized in groups: Pain Relief, ACE Inhibitors, Diuretics, Cardiovascular Drugs, Insulin or Antidiabetics, Muscle Relaxants, Psychoactive Drugs, Allopurinol, and Other. In addition, participants were asked to provide details of the provided health care and rehabilitation and to report what was most beneficial.

### Questionnaires

The following validated questionnaires were used: FSS (fatigue severity scale), WHODAS 2.0 (World Health Organization Disability Assessment Schedule), QoL/SF-36v2 (Quality of life/Short Form Health Survey) and IPAQ (International physical activity questionnaire). Data from the SF-36v2 was analyzed with Quality Metric Health Outcomes Scoring Software 5.0, data from the WHODAS 2.0 was converted to IRT (item-response-theory)-based scoring [6, 7] and data from the IPAQ was calculated to metabolic equivalent of task (MET)-min per week. [8–10]

We only used the data from the Dutch participants for the analyses of the SF-36v2 and IPAQ since the data of other countries were incomplete or not reliable. We refer to the results section for details of data.

### Data analysis

Mainly descriptive statistics were used to analyze the data and performed by using IBM SPSS Statistics 25. Absolute numbers and percentages of the number of answers on the item are presented. Correlations between age and disease severity were calculated (Pearson correlation coefficient). Analyses for the open-question sections were performed by narrative analysis using ATLAS.ti 2.0. Axial coding was used for the analyses to identify relations between the data and categorized when referring to the same phenomenon.

## Results

### Participants

In total 282 participants with confirmed diagnoses of muscle glycogenosis were included between 2015 and 2018. 269 (95%) of them had GSD5.

**Fig. 1:**
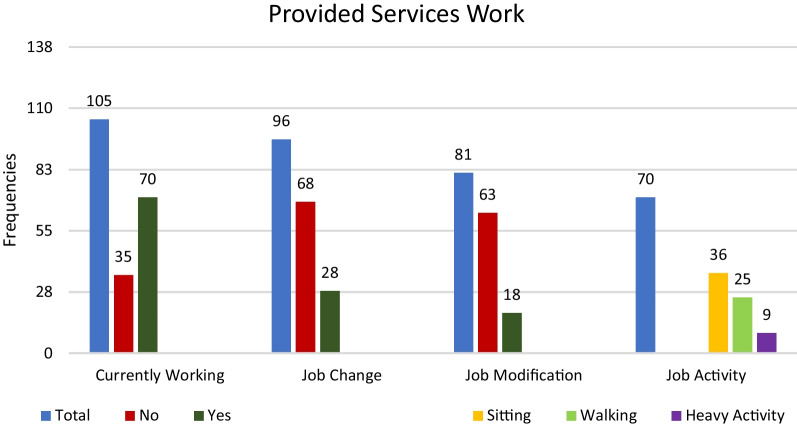
Work Involvement. ‘Total’ shows the number of answers for the question. Currently Working demonstrates how many people are currently working. Job Change demonstrates how many people have changed their job due to their disease (GSD5). Job Modification demonstrates for how many people their employer modified their work environment because of their disease conditions (GSD5). Job Activity demonstrates what people are doing most of their time during work

This report only presents the data of the participants with GSD5. Of the 269 GSD5 participants, 196 (73%) completed all mandatory questionnaires; for other participants, data were incomplete. Table 1 in Scalco et al. shows the completeness of data entry for different items [4]. The number of participants that have provided an answer to a question are provided in the tables and figures. Figure 1 in the first EUROMAC report by Scalco et al. [4] provides an overview of the geographical origin of the participants. The majority (215; 80%) were from the UK (United Kingdom), Spain, Italy or the Netherlands.

**Table 1:** Descriptive statistics on previous treatments

Previous ongoing treatments	Number of participants that responded	Frequency: number (% of total number of participants)
*Medication*		
Analgesic drugs	194	45 (23.2%)
ACE inhibitors	191	25 (13.1%)
Diuretics	195	10 (5.1%)
Cardiovascular	198	33 (16.7%)
Insulin and antidiabetics	194	9 (4.6%)
Muscle relaxants	189	8 (4.2%)
Psychoactive	192	34 (17.7%)
Allopurinol	187	9 (4.8%)

	Number of participants that responded	Frequency: number (% of total number of participants)	Total number of answers for beneficial question	Beneficial (% of participants who used this)
*Diet*				
Lipid rich	167	12 (7.2%)	11	5 (45.5%)
Carbohydrate Rich	174	29 (16.7%)	28	19 (67.9%)
Protein Rich	170	17 (10%)	16	5 (31.25%)
Low**-s**ucrose	173	55 (31.98%)	54	41 (75.9%)
Other diets: Fig. 3	17			
*Supplements*				
Vitamin B6	171	11 (6.4%)	11	2 (18.2%)
Creatine	172	11 (6.4%)	10	1 (10%)
BCAA*	170	3 (1.8%)	3	2 (66.7%)
Coqldebenone	172	0 (0%)	–	–
Carnitine	169	9 (5.3%)	9	0 (0%)
Other vitamins	173	15 (8.7%)	14	4 (28.6%)
Other supplements: Fig. 4	19			

*BCAA* branched-chain amino acids

### Work participation

Figure 1 provides an overview of the participants’ work involvement. Seventy (67%) participants reported to be working at the time of the study (0–20 years: 40% working; 20–60 years: 67% working and > 60 years: 32% working); the other 35 (33%) stated that they were not currently working. Participants were not asked if they had been working in the past. 28 (29%) of the respondents stated that they changed their job due to GSD5 and 18 (22%) needed their employer to modify their work environment because of their disease. Thirty-six (51%) participants reported that they were most of their time sitting during work, while 25 (36%) were mostly walking and 9 (13%) reported to have strenuous physical activities during work.

### Previous and ongoing treatments

**Fig. 2:**
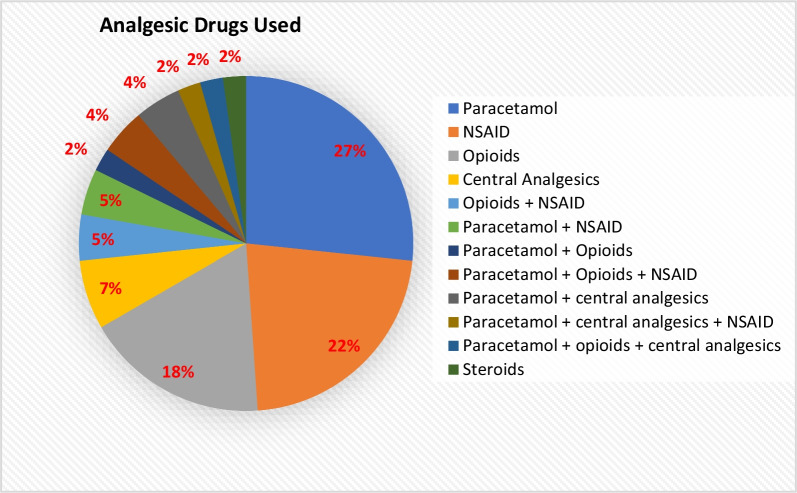
This Pie Chart demonstrates the types of pain medication that is being used by the participants as previous treatment for GSD5. It also shows the percentages per type of pain medication. Total number of answers: 45

Table 1 outlines which treatments the respondents had previously received of. Mostly used were medications for pain medication (n = 45; 23%), psychoactive drugs (n = 34; 18%), and cardiovascular drugs (n = 33; 17%). The types of pain medication that were used in this cohort are illustrated in Fig. 2. Paracetamol was used by 21 (47%) participants with or without other analgesic drugs, non-steroidal anti-inflammatory drugs (NSAID’s) by 10 (22%), and opioids by 8 (18%) of the participants. All 45 individuals who used pain-relief, indicated that they had done so to treat their GSD5 symptoms at some point in their lives.

**Fig. 3:**
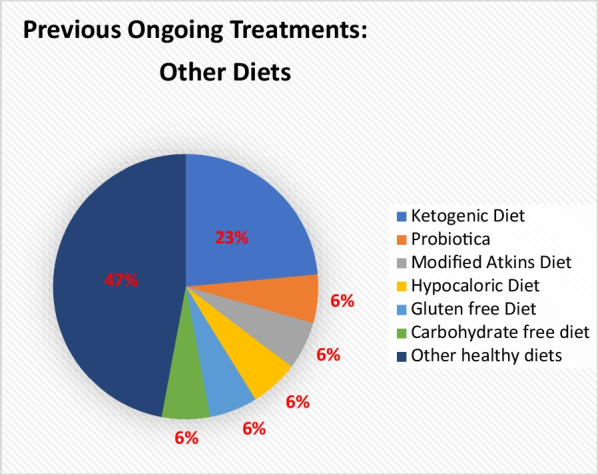
This pie chart demonstrates the mentioned ‘Other’ Diets in the open-question that have been used as a previous treatment. Ketogenic diet, modified Atkins and carbohydrate free are very similar, together 35%. Total number of answers: 17

Fifty-five participants (32%) had been on a low sucrose diet, and 41 (76%) of them found this diet beneficial for their GSD5-related symptoms. Twenty-nine (17%) had been on a carbohydrate-rich diet, and 19 (68%) of them reported a beneficial effect. A minority of the participants reported a positive effect of a protein-rich and lipid-rich diet. Other dietary interventions (n = 13) are illustrated in Fig. 3, including the ketogenic diet and other healthy diets. Of this group, 15 (88%) reported that those diets had positive/beneficial effect.

**Fig. 4:**
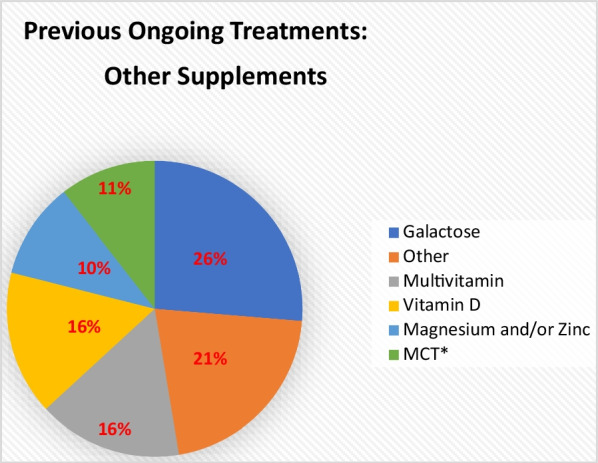
This pie chart demonstrates the mentioned ‘other’ supplements in the open-question that have been used as a previous treatment. Total number of answers: 19 *MCT: Medium-Chain-Triglyceride

Supplements had not been used by many participants. Only three participants had used branched chain amino acids (BCAA), two of whom reported it to be beneficial. Other supplements that had been used as a previous treatment by this cohort are illustrated in Fig. 4: galactose (n = 5; 26%), multivitamins (n = 3; 16%) and vitamin D (n = 3; 16%); 5 (26%) participants of this group reported a positive/beneficial effect.

### Health care and rehabilitation

Table 2 provides a summary of the types of rehabilitation program that were reported as previous ongoing treatments. The most frequently reported form of rehabilitation was walking (n = 69 (60%)). Physical therapy, muscle massage, working out in a gym and swimming also have been used more frequently than other programs.

**Table 2:** Program type of rehabilitation

Program type	Frequency: N (% of total (115))
Walking	69 (60%)
The gym and/or the pool	13 (11.3%)
Physical therapy	11 (9.6%)
Massage of muscles	11 (9.6%)
Physical therapy	11 (9.6%)
Occupational therapy and physical therapy	4 (3.5%)
Other exercises (e.g. stretching)	3 (2.6%)
Occupational therapy	2 (1.7%)
Aerobic training	1 (0.9%)

This table gives an overview of previous ongoing treatments, concerning rehabilitation. It is assessed with ATLAS.tii

Table 3 provides an overview of provided health care and rehabilitation. Physical therapy was reported to be mostly beneficial in the open questions (n = 10; 23%). Other beneficial approaches included receiving support for disabled workers, available literature and advice and the clinical assessments and follow-ups. Those types of health care have helped the participants with GSD5 related struggles.

**Table 3:** Health care details

Thematic category	Key terms (examples)	Reported (N total = 43):
Physical therapy	Second wind, motor function assessment, stress test, cramps massage, aerobic training	10
Literature, advice, information	Books (Wakelin), advice from Neurologist and other specialists	6
Clinical assessment and follow-up	–	6
Support for disabled worker	Parking card, disability pension, support for all expenses	6
Diagnosis	Understanding disease and cause of symptoms, prevent rhabdomyolysis	4
Occupational therapy	Balance activities and abilities, stimulation exercise, dealing with limitations, advices	4
Exercise	Walking, daily movement, electric bike, scoot mobile, sport	4
Diet	Minimal sugar, more proteins and fat	2
Cognitive behavioral therapy	–	1

This table gives an overview of the mentioned types of health care and their value to the participants. It is assessed with ATLAS.tii

The limitations the participants with GSD5 reported to experience in their everyday life are presented in Table 4. Almost all (n = 79; 93%) experienced limitations in climbing stairs. Also, limitations in running (n = 76; 89%), walking fast (n = 72; 85%) and carrying shopping bags (n = 69; 81%) were frequently reported.

**Table 4:** Limitations

Limitations	N = (% of total (85))
Climbing stairs	79 (93%)
Running	76 (89%)
Walking fast	72 (85%)
Carrying or holding shopping bags	69 (81%)
Taking care of household	64 (75%)
Biking	63 (74%)
Chewing	62 (73%)
Standing up for a long time	62 (73%)
Washing your body	58 (68%)
Brushing your hair	58 (68%)
Rising from the floor	58 (68%)
Remembering things	57 (67%)
Getting dressed	56 (66%)
Opening jars	56 (66%)
Rising from a chair	56 (66%)
Eating	55 (65%)
Driving a car	51 (60%)
Sex	51 (60%)

### Validated questionnaires

The results of the QoL, FSS, IPAQ and WHODAS are included in Table 5**.** We excluded data of the QoL, IPAQ and WHODAS from unrealistic (outside the range, below or above the standardized limit) and/or incomplete scores, resulting in the inclusion between 21 and 34 responses. For the analyses of the QoL we only used data from the Dutch sub cohort, since this data set was complete and accurate. Other sub-cohorts did not include any realistic or complete scores.

**Table 5:** Descriptive statistics on validated questionnaires

Questionnaire (range)	Mean (SD)	Median	N	Interval (min–max)
Fatigue Severity Scale (Total: (9–63) ÷ 9 = 1–7)	46.05 (13.6) 5.12 (1.5)	47.0 5.22	34	[15.0;63.0]
WHO Disability Assessment Schedule 2.0 (0–100)	13.5 (13.0)	10.0	27	[1.0;43.0]
Short Form 36 Health Survey (16–112)	49.5 (22.4)	60	21	[16.0;80.0]
International Physical Activity Questionnaire (Category Low, Moderate or High in MET-min per week)	7257.3 (9423.3)	2491.5	26	[0.0;38,681.0]

This table shows the results of the validated questionnaires

*Fatigue.* The median for the FSS was 5.22 [1.7;7]. Twenty-nine out of 34 participants had a FSS score of ≥ 4. Since a score of ≥ 4 generally indicates severe fatigue [11], this sample is considered to be severely fatigued.

#### Quality of life

The median for the total score of the SF-36 is 60 [16.0;80.0]. Higher scores indicate a higher quality of life. Compared to healthy adolescents the scores of the QoL for GSD5 participants were well below the normal range, especially the score of the physical functioning (PF) which was the lowest of all measures (44,8).

*Disability.* The median IRT-based range score for the WHODAS 2.0, is 10 [1.0;43.0]. The higher the domain score, the higher the level of experienced disability. The correlation between age and disability (measured by WHODAS) was analyzed, and due to the significance level being above the threshold (p < 0.05), this correlation is not considered statistically significant.

#### Physical activity

The median score on the IPAQ was 2491 [0.0;38,681.0]. MET-min/week and 20 out of 26 participants scored a low physical activity. With the calculated MET-min per week we can conclude that this sample scored a low physical activity since the score did not exceed the minimum criteria of 3000 METs. [9, 10]

## Discussion

The EUROMAC registry was created to ensure data collection and its maintenance with the aim to improve diagnosis and care for individuals with rare glycogen storage diseases. The registry increases knowledge from clinical data from individuals with GSD5 and related conditions and has played a positive role in promoting translational research [3]. Moreover, it is one of few international registry of muscle glycogenosis available. Besides unraveling phenotypic characteristics of GSD5 as seen in the accompanying papers [3, 4], the current report provides insight in the previous treatments, functional limitations, fatigue, health disability, quality of life and physical activity. We will discuss the main findings below.

The number of participants currently working (67%) is in line with the proportion of the general adult population that is working in various European countries: 72% in the Netherlands [13], 76% in the UK [25], and 66% in Spain [26]. This normal labor force participation rate might be related to the reported adaptations: 29% had changed their job and 22% had their work environment modified. Reported physical activity during work is variable, including sitting and non-sitting labor.

In line with previous studies, many participants reported use of medication to relieve pain. Both over the counter and prescribed drugs were used, mostly paracetamol (acetaminophen). Paracetamol is unlikely to relieve acute muscle pain but may be taken to manage more severe pain, which may last for hours after exercise [14]. Furthermore, 26% of participants reported opioid medication in combination with other analgesic drugs. This is not recommended, as these drugs may mask feedback from muscles, leading to further muscle damage and recurring pain [14]. The recently published Clinical Practice Guidelines recommend a regular exercise program as a safe manner to reduce chronic pain [14–17]. Other drugs commonly used are psychoactive drugs (18% compared to 5.5% in general population) and cardiovascular drugs (17% compared to), which make sense since a high rate of coronary heart disease was described in the EUROMAC paper [4]. For comparison, 10.7% of U.S. adults used one or more prescribed pain medications in the past 30 days; 13% of males use over the counter analgesics weekly and 11.9% of US adults reported having used opioids in the past 12 months (data mainly from USA, 2015–2018) [32–34].

Most of the participants who used a sucrose supplementation and carbohydrate rich diet found it beneficial. The beneficial effects of oral sucrose supplementation before exercise are well established [14, 19, 29]. Also, a carbohydrate rich diet has shown to attenuate muscle pain in the first few minutes of exercise before the second wind compared to a protein rich diet and has proven beneficial because it maintains the hepatic glycogen stores [14, 20, 21]. Both the protein-rich and the lipid-rich diet were reported as not beneficial for our cohort but only by a low sample size. Still, improvement in parameters for the sucrose supplementation and carbohydrate rich diet need to be studied since none indicated significant clinical benefits.

In the open-questions the ketogenic diet was reported as beneficial. Other studies that have investigated the effects of a modified ketogenic diet in individuals with GSD5 and one informal survey showed improvement in symptoms and exercise tolerance for most of the cohort after using the ketogenic diet. [22, 23] The BCAA supplementation was found beneficial for 67% but since only 3 participants have used it, not much can be concluded.

The reported rehabilitation programs are in line with recommended physical and occupational therapy. Remarkably, 60% of the participants reported walking as an important rehabilitation approach. The international patient support group IamGSD has promoted walking as a training for GSD5 for years [24]. Also, walking can easily be implemented in daily life. Future research could focus on objectively demonstrating the positive effect of walking and other forms of aerobic exercise in daily life. The inclusion of participants in the registry may have resulted in a higher proportion of individuals who are more active, presumably both mentally and physically, as they actively seek medical assistance. However, caution should be exercised due to the potential overestimation of rehabilitation use stemming from selection bias.

Moreover, for comparison of fatigue, the FSS score for individuals with Lyme disease is 4.8, Parkinson’s disease is 4.1 and for a healthy population it is 2.3. [11, 12, 30]

Limitations of this study include the collection of retrospective data and the issue of missing data, which reduces the validity. The data was collected in the prospective EUROMAC registry and had to be entered by various researchers and clinicians. This carries the risk of observation or confirmation bias in case of subjective outcome measures. The data from the WHODAS was not of sufficient quality to be interpreted further. It is also important to note that the present study had a limited number of correlation analyses, which may restrict the generalizability of the findings and the ability to explore potential relationships between additional variables of interest. Furthermore, to have researchers and clinicians enter the patient-reported outcome measures scores was challenging. It has led to missing data and implies propensity to error. Prospective patient registries where patient-reported outcome measures (PROMS) are completed by patients themselves are expected to have a better validity [27, 28]. Nevertheless, the EUROMAC has raised awareness of diagnostic accuracy and collected important data for use in future clinical trials with the accompanying papers [3, 4].

## Conclusions

The EUROMAC registry helps to gain more understanding of GSD5. It provides inspiration for other clinicians to develop patient registries for other rare diseases. The analyses in this paper has given insight into the functional limitations and treatments and rehabilitation approaches used. The findings support the recent international recommendations for people with GSD5, including adopting an active, healthy lifestyle and careful use of analgesic drugs. Moreover, participants that used diet and supplementation mentioned beneficial effects. This can be promising, however placebo-controlled studies with larger cohorts are warranted to provide conclusive evidence of the effects of these diets and supplementations.

## Data Availability

Only data that is relevant and not analyzed before is included in this article. Other data are reported in the accompanied paper by Scalco et al. in 2020 in this journal [4]. The database is available upon reasonable request to the EUROMAC consortium.
